# Heart Valve Disease: Challenges and New Opportunities

**DOI:** 10.3389/fcvm.2020.602271

**Published:** 2020-10-22

**Authors:** Francesca Bartoli-Leonard, Elena Aikawa

**Affiliations:** ^1^Division of Cardiovascular Medicine, Department of Medicine, Center for Interdisciplinary Cardiovascular Sciences, Harvard Medical School, Brigham and Women's Hospital, Boston, MA, United States; ^2^Division of Cardiovascular Medicine, Department of Medicine, Center for Excellence in Vascular Biology, Harvard Medical School, Brigham and Women's Hospital, Boston, MA, United States; ^3^Department of Human Pathology, Sechenov First Moscow State Medical University, Moscow, Russia

**Keywords:** heart valve disease, calcification, drug discovery, statins, tissue engineered heart valves (TEHV)

Heart valve disease, including congenital, rheumatic, degenerative and calcific now occupies centre stage in the field of cardiovascular medicine. As the worldwide population ages and prevalence of degenerative diseases increases, advances in imaging and biomarker recognition has catapulted this once unappreciated pathology into the minds of clinicians, researchers and engineers alike. Whilst challenges such as diversity in clinical research and understanding how best to achieve global dissemination of science must remain at the forefront of our goals, advances in novel technologies and integrated computational approaches have opened up this field to exciting new opportunities for greater understanding within this complex but undervalued subject area. Frontiers in Cardiovascular Medicine has now recognised the ever-growing importance of heart valve disease, and this grand challenge must be met in uncountable ways if we are to succeed in tackling this widespread problem head on in the coming years.

## Challenges in Cardiovascular Imaging for Early Disease Detection

Since Braunwald's seminal paper (1) outlining the history and pathology of valve disease, prevalence within the western world has inflated to in-excess of 30 million people living with heart valve disease, with incidences increasing with age (2). Aortic stenosis represents the clinical manifestation of calcific aortic valve disease, in which remodeling of the valvular tissue results in severe hemodynamic changes within the valves due to formation of fibrocalcific nodules (3). Current diagnostic tools focus on qualitative measurements of aortic valve thickening via echocardiography or computed tomography imaging (4), both of which give limited insight into the pathophysiology and poorly predict early stage disease progression (e.g., formation of microcalcification), stressing the need for the ability to stratify patients before intervention. Advances in preclinical near-infrared fluorescence imaging has enabled simultaneous *in vivo* visualization of early mineralization, however the processes of microcalcification formation, vesicle release, aggregation and nucleation, are still far beyond the resolution for current imaging modalities. Within the last decade duplexing positron emission tomography (PET) tracers has allowed for some success in early detection of calcification in patient with calcific aortic valve disease (5), however, with no large clinical trials currently in progress, this diagnostic technique is still under validation and the need for early detection of disease is still apparent. Moreover, as statin trials show no benefit in treatment of aortic stenosis, and therapy for valve disease continues to lag behind the majority of other cardiovascular disorders, the mainstay of treatment remains invasive and costly surgery in the form of a mechanical or bioprosthetic valve replacement or transcatheter aortic valve implantation. However, as both these interventions are performed at relatively late stages of disease the procedure is often accompanied with excessive operative risk and with repeat surgery often needed throughout the patient's life, thus highlighting the urgent need for non-invasive treatments.

## Challenges in Valve Replacement and the Development of Tissue Engineered Heart Valves

Due to limitations of mechanical and bioprosthetic valves, much of the current focus of cardiovascular scientists and bioengineers remains on tissue engineered heart valves; valves which can incorporate biomaterials with autologous cells, allowing the tissue construct to be completely biocompatible. While xenograft heart valves have been used for the last 50 years, issues remain with tissue durability and the formation of calcification potentially due to the elicited immune response (6). Since bioprosthetic valves are currently widely used for surgical and transcatheter valve replacement, the need to overcome tissue calcification is urgent. Genetically engineered pigs may provide the ideal heart valve, however recent advances within the stem cell field are paving the way to the possible use of iPSCs captured in 3D hydrogels (7), which would not only provide a suitable cell source for individual patients, but with the ability to predetermine the starting matrix composition may provide a suitable substrate, which can degrade as the cells produce their own extracellular matrix. Unfortunately, tissue engineered heart valves still remain an elusive pipedream for many patients, who are left facing a lifelong management with anticoagulant medication and extensive treatment. Thus, with current advancements in bench-top research hoping to address the limitations currently faced today, the future in which lab grown valve replacements could become a not too distant reality.

## Challenges in Pharmacological Innovation and Personalized Medicine

In leu of an autologous replacement, pharmacological treatment of heart valve disease is lacking, with no rigorous evidence supporting any pharmacological intervention in acute valvular disease. However, some medications may be used as a way in which to delay surgery, promote cardiac function or reduce valve inflammation. The first prospective study to evaluate statins in aortic stenosis; Rosuvastatin Affecting Aortic Valve Endothelium to Slow the Progression of Aortic Stenosis (RAAVE), suggested that asymptomatic patients with moderate to severe stenosis benefited from treatment, with a reduction seen in the increase of stenosis measured by echocardiogram (8). Conversely, the Scottish Aortic Stenosis and Lipid Lowering Trial, Impact of Regression (SALTIRE) study (9), conducted around the same time, found no significant effect following 25 months of treatment, ultimately leading to discordance within the field on the efficacy of statins in heart valve disease. A multitude of reasons could explain the lack of patient response to statins. Within atherosclerotic patients, the majority of benefits reported from statin intervention arises from a reduction in macrophage driven inflammation, reducing the overall cytokine production within the vessels. This allows for significant cell substrate to be deposited within the vessel in the form of collagen accumulation and reduction of matrix degradation, leading to a more stable fibrous cap and a further reduction in inflammation. Conversely, in valve disease, accumulation of collagen leads to valve thickening, increased fibrosis and reduction of overall cardiac output (10). Moreover, statins are often used too late within patient care for valve disease (11), often once the leaflet biomechanics have been affected, bringing rise to the question whether statins do not work in valve disease patients, or are administered past the “point of no return” in the majority of cases. If patients need to be better stratified in order to respond to statins, or if multiple medications need to be administered at the same time in order to be of benefit, the specific mode of action of statins must be fully understood before they can be considered an effective treatment for heart valve disease.

The other pharmacological treatment which has gripped the valve field more recently is proprotein convertase kexin 9 (PCSK9) inhibitors, developed primarily for the treatment of hypercholesterolemia, and have been shown to be more effective than statin therapy in decreasing both Lipoprotein(a) [Lp(a)] and low-density lipoprotein cholesterol (11), both of which are linked to the development of valve disease and calcific stenosis. With recent studies highlighting the potential role of PCSK9 inhibitors on the etiology of valve disease (12) the lack of current clinical trials investigating the reduction of associated valvular outcomes remains an obstacle to face before PCSK9 inhibition is widely used within the clinic. Nevertheless, 50 years on from Braunwald's pivotal paper, the Further Cardiovascular Outcomes Research with PCSK9 Inhibition in Subjects with Elevated Risk (FOURIER) trial (13) conducted at Brigham and Woman's Hospital, has revealed that PCSK9 inhibition with evolocumab could decrease calcific aortic valve disease incidence in patients with cardiovascular disease, highlighting the desperate need for more focused trials on this emerging line of treatment. To date, the Aikawa lab at the same institution is continuing this pioneering research on heart valve disease, including recent work demonstrating the role of PCSK9 in development of cardiovascular calcification in murine models (14), highlighting the need for more translational research to truly understand this and other therapeutic interventions. Thus far, no drug has been specifically developed for the use in chronic heart valve disease, and while every effort has been made to repurpose drug therapies to address this, until more is known about the pathobiology of valve disease development, future trials must be focused purely on negating the end stage events of heart valve disease.

## Challenges in Mechanistic Understanding and Basic Research Translation

**Figure 1:**
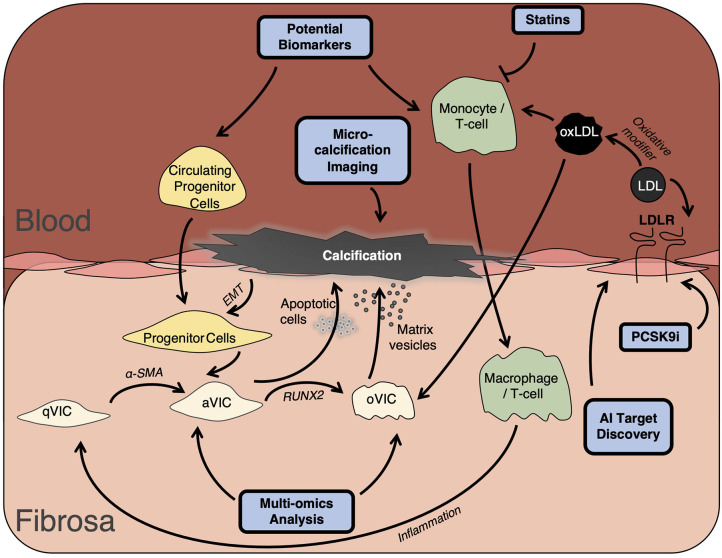
Development of valve disease. Development of valve disease occurs following prolonged inflammation driven by macrophage and leukocyte infiltration and via VIC differentiation through accumulation of apoptotic bodies and calcific matrix vesicles. Ultimately resulting in collagen remodeling, leaflet thickening, and valvular calcification. qVIC, quiescent valve interstitial cell; aVIC, activated valve interstitial cell; oVIC, osteogenic valve interstitial cell; EMT, endothelial-to-mesenchymal transition; LDL, low-density lipoprotein; RUNX2, Runt related transcription factor; PCSK9i, proprotein convertase subtilisin/kexin type 9 inhibitor; AI, Artificial Intelligence.

As precision medicine moves to the forefront of patient care, researchers and clinicians must find the potential upstream biomarkers before the onset of valve disease. Delineating the underlying mechanism of the phenotypic switch from healthy valve cells to pro-inflammatory and calcific phenotypes still eludes researchers today, with the true cause of this differentiation still not fully understood. Moreover, it is still unknown whether valvular interstitial cells are in fact representing a continuing spectrum of cells differentiating from fibroblasts to myofibroblasts and onto osteogenic cells, or whether there is a subpopulation of resident progenitor cells contributing to osteogenesis (Figure 1). While the capacity to differentiate and produce a mineralized matrix is now considered an inherent property of valvular cells (3), the regulation of this process is controlled by a wide range of factors, from Wnt signaling to low-density lipoprotein and inflammatory cytokines, with the true answer laying somewhere closer to an amalgamation of all of the above and more. One such biomarker, Lp(a) has been at the forefront of these risk factors, associated with cardiovascular disease independent of other traditional risk factors. The lipoprotein has been demonstrated to be associated with calcific aortic valve disease as far back as 1995 (15), with strong association in asymptotic patients before they present in the clinic. Thus, Lp(a) presents a new cohort of therapeutic targets, with greater impetus on research to unpick the mechanism and mediators of increased Lp(a) expression in preclinical patients. Another such interesting avenue of future work; extracellular vesicles, have been shown to mediate atherosclerotic plaque progression (16, 17), and with their contribution to microcalcification known, could also be the driving force behind aortic valve disease. Recent work has implicated extracellular vesicles as important drivers of mineralization, depositing their bioactive cargo and mediating cell-cell communication throughout the vasculature, suggesting a possible shared driver between these two pathologies. To this end, multi-omic approaches have sought to expedite the progress in the field though networks and systems biology, through identifying the molecular networks in valve disease and producing novel targets for drug discovery and development (18). It is important to note however, that whilst valve disease which often generalized as one disease, is in fact multifaceted and varied, arising from a multitude of distinct pathologies from chronic kidney disease to systemic lupus erythematosus. Until standardization of the field is reached, stratification of treatment may be unobtainable (19).

## Clinical Research and Diversity

Despite the aforementioned breakthroughs in treatment and patient diagnosis, much of the current research focuses solely on male heart valves, more specifically, Caucasians. Whilst the NIH revitalization act of 1993 required the inclusion of woman in NIH-funded clinical research, it was not until 2015 that the NIH announced policies requiring sex as a biological variable in study design and reporting. With growing interest on the role of sex hormones, biological sex and race in cardiovascular medicine and the startling difference in progression of heart disease between these variables (20) a need for more diversity in research studies has never been more apparent.

## Opportunities and Future Perspectives

The grand challenge of heart valve disease is much like heart valve disease itself, multifaceted and complex, yet with the right tools, solvable. Valve disease is a preventable pathology and with the current advances in modern medicine moving forward, should become treatable. Over the past decade the field has seen an explosion of research centered upon less invasive valve procedures utilizing transcatheter approaches. While this procedure has revolutionized the field, the implanted valves are still suffering inherited problems associated with bioprosthetic valve durability. Undoubtably, the greatest challenge still remains in early detection of valve disease, required to stratify patients quickly and effectively, tailoring the correct treatment to the patient immediately, thus increasing the chance of a full recovery. Whilst there are a number of barriers still to overcome in treatment for valve disease, they also represent opportunities to push forward in patient centered research, through collaboration and cross-disciplinary studies. Moreover, as clinicians and scientists we must truly understand the molecular signature of heart valve disease and distinguish it from the menagerie of complex cardiovascular pathologies. The overreaching goal of this Frontiers in Cardiovascular Medicine section in Heart Valve Disease is to provide an open forum to comprehend the ever-changing landscape of valve research and constructively increase the knowledge within the field. We expect contributions from clinical and basic scientists to this grand challenge as it is only through incorporating multidisciplinary approaches with novel techniques for drug discovery such as artificial intelligence, bioinformatics and computational science that we will be able to truly understand and treat this devastating pathology.

## Author Contributions

All authors listed have made a substantial, direct and intellectual contribution to the work, and approved it for publication.

## Conflict of Interest

The authors declare that the research was conducted in the absence of any commercial or financial relationships that could be construed as a potential conflict of interest.
